# Solitary Plasmacytoma of the Mandible: A Rare Presentation

**DOI:** 10.7759/cureus.109034

**Published:** 2026-05-17

**Authors:** Sayan Chattopadhyay, Narendra Prakash Rai, Rachita Arora, Sumit Dutta, Snehanjan Sarangi

**Affiliations:** 1 Oral Medicine and Radiology, Dr. R. Ahmed Dental College and Hospital, Kolkata, IND; 2 Oral Medicine and Radiology, Faculty of Dentistry, Malaysian Allied Health Sciences Academy (MAHSA), Petaling Jaya, MYS; 3 Pediatric and Preventive Dentistry, Dr. R. Ahmed Dental College and Hospital, Kolkata, IND; 4 Oral Medicine and Radiology, Kalka Dental College, Meerut, IND; 5 Oral and Maxillofacial Pathology, Dr. R. Ahmed Dental College and Hospital, Kolkata, IND

**Keywords:** lymphoid malignancies, mandible, plasma cell neoplasm, radiation therapy, solitary plasmacytoma

## Abstract

Solitary plasmacytoma is a rare plasma cell neoplasm, most often arising in vertebrae and long bones; among these, mandibular involvement is exceptional. The following report documents a rare mandibular presentation in a 48‑year‑old female patient who presented with pain and swelling in the right lower jaw. Clinical examination, radiographic findings, and histopathology confirmed the diagnosis. The lesion was managed surgically with hemi‑mandibulectomy and reconstruction, with no recurrence to date.

This case highlights the importance of early recognition of maxillofacial plasmacytomas by dental practitioners to enable timely diagnosis and improve treatment outcomes.

## Introduction

Plasmacytoma is a malignant tumor arising from plasma cells, which may develop either within soft tissues or in the axial skeleton. It exists in localized forms, namely solitary bone plasmacytoma (SBP) and extramedullary plasmacytoma (EMP) [1,2]. Unlike SBP, EMP develops in soft tissues. It has a better prognosis than SBP, which has a greater chance of transforming into multiple myeloma, the disseminated form of plasmacytoma [3]. SBP develops when abnormal plasma cells within the bone marrow accumulate and give rise to a single localized tumor in a bone [3]. The long bones and the vertebrae are usually affected. SBP involving the jaws is an uncommon presentation, typically appearing as a single osteolytic lesion in the absence of generalized bone marrow plasmacytosis, and accounts for nearly 3% of all plasma cell neoplastic lesions [4]. The mandible is more frequently affected than the maxilla, with the local bone pain and lesion being confined to the alveolar ridge. It usually has a male predilection occurring in the sixth to seventh decades of life [5,6]. On a random marrow sample, the absence of clonal plasma cells is detected. Solitary plasmacytoma must be distinguished from multiple myeloma because SBP represents a localized, potentially curable plasma cell neoplasm confined to a single bone lesion without systemic involvement, whereas multiple myeloma is a disseminated malignancy with clonal plasma cells in bone marrow, multiple lytic lesions, M-proteinemia, and the presence of hypercalcemia, renal failure, anemia, and bone lesions, requiring entirely different management (local RT vs. systemic chemotherapy) [7]. Mandibular SBP clinically mimics odontogenic lesions (cysts, ameloblastoma) due to well-defined radiolucencies in the posterior body/angle, often with tooth mobility/swelling but without pulp vitality changes, leading to misdiagnosis and inappropriate conservative management [8].

Early recognition by dental practitioners is crucial as delayed biopsy risks pathologic fracture, inferior alveolar nerve damage, or progression to myeloma (10-30% within 5 years), while prompt multidisciplinary referral enables definitive RT/surgery with >80% local control. Presence of M-protein (monoclonal immunoglobulin) in serum and urine and bone pain, anemia, hypercalcemia, thrombocytopenia, neutropenia, renal failure together suggest systemic myeloma [5,6,9].

## Case presentation

A 48-year-old woman presented to the department with complaints of pain and swelling in the right lower side of her face for 2-3 months. The pain was localized, gradual in onset, and intermittent in nature, wherein the patient complained of occasional pain localized in the right side of the mandible. Initially, the swelling was smaller in size, gradually expanding to attain the present size. No similar swellings were seen elsewhere in the body, and there were no associated symptoms. Extraorally, the swelling extended superoinferiorly from the ramus to the body of the right mandible. It was 6x5 cm, roughly oval in shape, with a smooth overlying surface, and normal surrounding mucosa (Figure 1a-1b).

**Figure 1:**
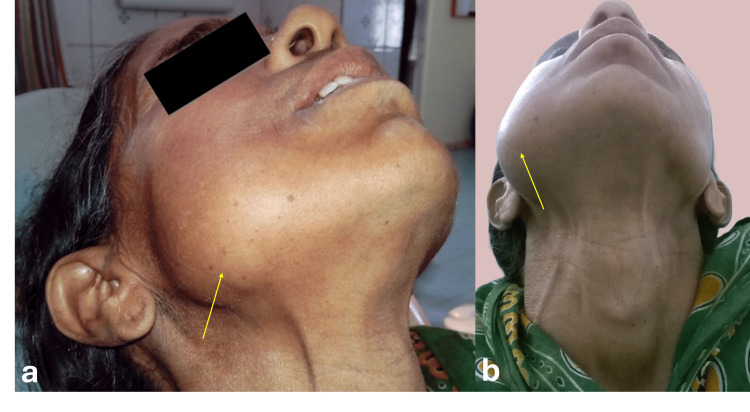
Extraoral clinical photographs A diffuse swelling over the right side of the mandible. (a) lateral view (b) inferior view

On palpation, the swelling was firm to hard in consistency. There was no bleeding, no discharge, and it was not fixed to the underlying structures. No regional lymphadenopathy was observed. Intraorally, a partially edentulous area was observed in relation to 46, with a healing extracted socket region observed in the same area. A grossly decayed tooth was seen in relation to 48, with a disto-proximal carious lesion observed in relation to 47. Grade I mobility was observed in 47 and 48. Surface area ulceration over the swelling was absent. Vestibular obliteration was also observed in the right posterior mandible. The swelling was associated with paresthesia of the right lower lip. A provisional diagnosis of radicular cyst was given due to a prior history of decayed tooth and extraction in the same site. A differential diagnosis of residual cyst, granuloma, and ameloblastoma was considered. A panoramic radiograph was obtained, which revealed a diffuse radiolucency, extending from the mesial of 47 to the distal aspect of 48, conforming to the ascending border of ramus of the right mandible (Figure 2).

**Figure 2:**
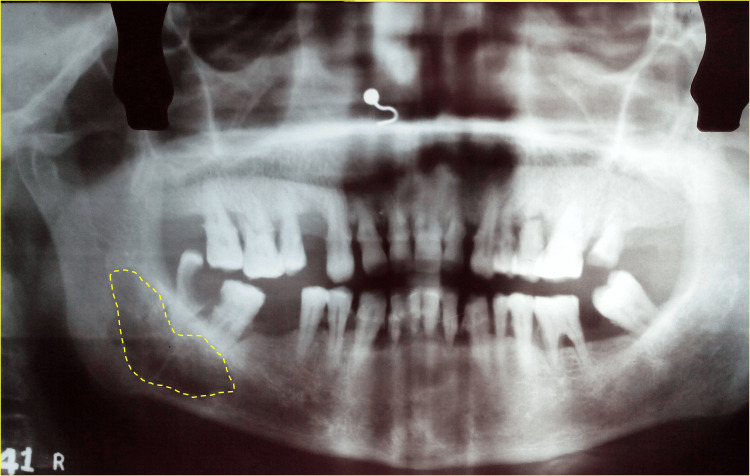
Panoramic radiograph A poorly defined radiolucent lesion in the right posterior mandible (outlined by yellow dotted arrows), extending from the molar region to the ramus.

Further systemic investigations, including serum/urine protein electrophoresis with immunofixation, CBC, and renal function tests, were performed. The absence of monoclonal protein, significant urine light chains, anemia, and a normal serum free light chains (sFLC) ratio indicated the absence of malignant myeloma and a lower risk of progression. Incisional biopsy was performed, and the specimen was sent for histopathological examination. Histopathology showed atypical plasmacytoid cells with abundant cytoplasm and nucleus pushed towards the periphery, arranged in a sheet-like manner (green arrow). Immunohistochemical staining showed strongly diffuse immunohistochemical positivity noted for CD138 (blue arrow) (Figure 3A-3D). 

**Figure 3:**
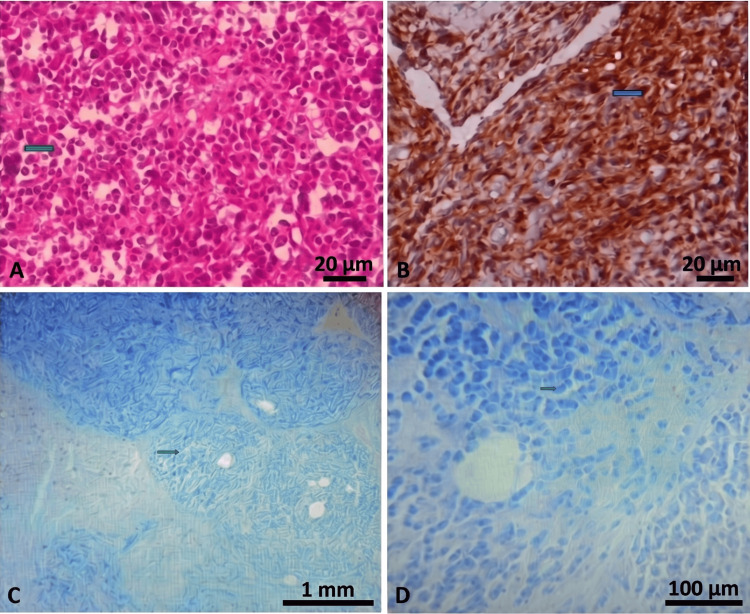
Histopathology and IHC with appropriate scale bars A: Hematoxylin and eosin-stained section (40x magnification) showing atypical plasmacytoid cells with abundant cytoplasm and nucleus pushed towards the periphery, arranged in a sheet-like manner (green arrow). B: Immunohistochemical staining (40x magnification). Strongly diffuse immuno-histochemical positivity noted for CD138 (blue arrow). C: Hematoxylin and eosin-stained section (10x magnification) showing atypical plasmacytoid cells with abundant cytoplasm and nucleus pushed towards the periphery (green arrow). D: Hematoxylin and eosin-stained section (40x magnification). Dense sheets of atypical plasmacytoid cells in a background of fibro-collagenous stroma (green arrow).

The lesion was surgically excised along with hemi-mandibulectomy, followed by reconstruction (Figure 4). The patient was followed up at 1, 3, and 8 months post‑surgery, with all visits showing satisfactory healing and no evidence of recurrence.

**Figure 4:**
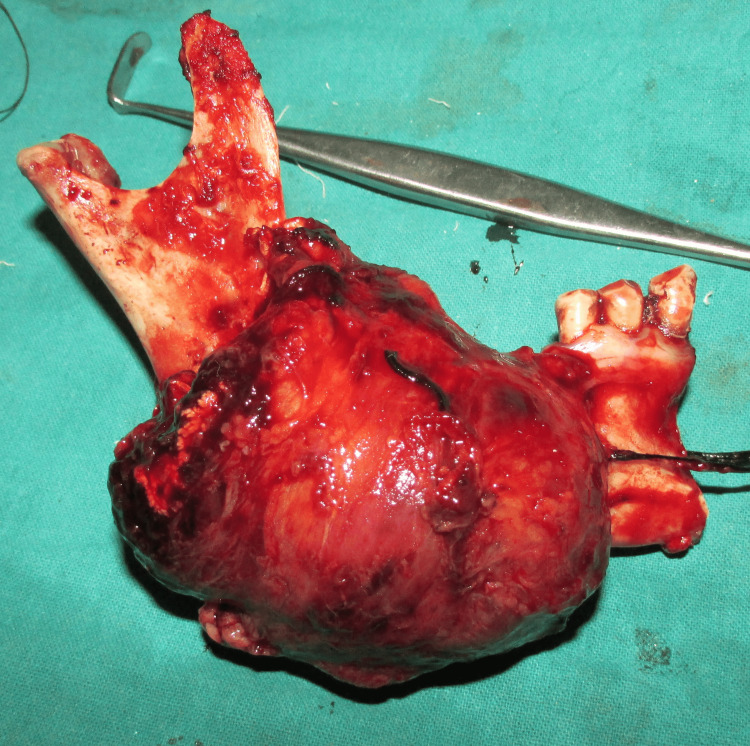
Surgical specimen following hemi-mandibulectomy The resected segment of the mandible with the associated tumor mass.

## Discussion

Early reports in 1846 by Dalrymple and Bence Jones described the features associated with the neoplastic proliferation of plasma cells [9]. SBPs usually affect bones of the vertebrae, ribs, pelvis, and pectoral girdle, while extramedullary plasmacytomas usually appear on the mucosal surfaces, e.g., nasal sinuses, oropharynx, larynx, etc. SBP is a disorder of plasma cell origin in which a single localized mass develops within osseous tissue. It arises due to the focal proliferation of atypical plasma cells derived from the bone marrow, which gradually accumulate along the inner aspect of the bone [10]. Cases of SBP affecting the head and neck are infrequently encountered, and usually the sinonasal tract is more involved than other bones. Korolkowa et al. found that the occurrence of plasmacytomas in the nasal cavity and paranasal sinus was 40%, 20% in the nasopharynx, and 18% in the oropharynx [11]. Clinical features may include pain involving the jaws or teeth, sensory disturbances such as paresthesia or anesthesia, loosening and displacement of teeth, episodes of bleeding, swelling affecting both hard and soft tissues, and, in advanced cases, pathological fractures in the affected areas. Fever and fatigue are some of the systemic manifestations. Its peak incidence is during the 6th and 7th decades of life with a male predilection, the ratio being 7:1 [5,6].

In our case, for the SBP of the jaw in our patient, hemi-mandibulectomy was selected over primary radiotherapy (RT) or combined modality due to the lesion's aggressive bone destruction compromising mandibular integrity from body to ramus/third molar region, necessitating complete resection for free histologic margins, aesthetic reconstruction, and functional preservation. While RT alone achieves >80% local control for radiosensitive SBP and is preferred per IMWG guidelines to avoid mutilation, jaw-specific cases often mandate surgery when tumors exceed 4-5 cm, exhibit partial RT response, or threaten structural stability, particularly here, where prior conservative observation or RT might risk pathologic fracture. Systemic workup confirmed true SBP, obviating systemic therapy, while hemi-mandibulectomy enabled immediate reconstruction sans adjuvant RT given clean margins, aligning with published case series where mandibular SBP resection outperforms RT for locoregional control in advanced presentations. No neurologic sequelae (paresthesia absent) further supported conservative excision over RT-induced fibrosis risks [8,12].

Lae et al. showed the maxilla being the most common site with female predilection rather than males [6]. A higher occurrence rate has been observed among individuals of African-American origin, with the incidence of plasma cell tumors ranging from 2.6 to 3.3 per lakh population [13,14]. Till now, a very limited number of cases of solitary plasmacytoma have been reported in the literature. Proliferation of plasma cells leads to the development of plasma cell neoplasms, which are similar to myeloma cells cytologically and immunophenotypically. Since these plasma cells acquire osteoclast-activating factors, this leads to the development of a unilocular or multilocular radiolucency due to resorption of the bone. Clinically, skeletal plasmacytomas appear to have radiolytic lesions involving any part of the skeleton. Radiographic findings are generally found in the apical areas of teeth [15]. Solitary plasmacytomas respond well to radiation therapy, so the treatment plan can be based on surgery alone, radiation therapy, or a combination of both as primary treatment modalities, depending on the size and site of the lesion. Radiation doses of 40-50 Gy are seen to be locally effective in 80% of the cases [16]. When whole tumour removal needs to be done, then we should opt for surgical removal with the aim of limiting cosmetic and functional compromise, particularly in cases with suspected pathological fracture. Ongoing investigations are focusing on the potential of anti-angiogenic agents such as thalidomide, along with proteasome inhibitors, as other treatment modalities in cases with plasma cell neoplasms [15]. Besides, long-term surveillance is mandatory given 10-30% progression to multiple myeloma within 5 years and 50-60% 10-year local control rates with contemporary management [17].

## Conclusions

Solitary plasmacytoma of the jaw is an uncommon plasma cell tumour that can mimic odontogenic lesions clinically. Histopathological and immunohistochemical assessment is important for correct diagnosis and suitable therapy. Early detection and long-term follow-up are critical because of the possible risk of progression to multiple myeloma.
